# Body Weight and Metabolic Rate Changes in Narcolepsy: Current Knowledge and Future Directions

**DOI:** 10.3390/metabo12111120

**Published:** 2022-11-16

**Authors:** Hamza O. Dhafar, Ahmed S. BaHammam

**Affiliations:** 1 The University Sleep Disorders Center, Department of Medicine, College of Medicine, King Saud University, Riyadh 11451, Saudi Arabia; 2 Department of Family Medicine, Prince Mansour Military Hospital, Taif 26526, Saudi Arabia; 3 The Strategic Technologies Program of the National Plan for Sciences and Technology and Innovation in the Kingdom of Saudi Arabia, P.O. Box 2454, Riyadh 11324, Saudi Arabia

**Keywords:** metabolic rate, energy expenditure, hypocretin, eating behavior, exercise, obesity

## Abstract

Narcolepsy is a known auto-immune disease that presents mainly in the teenage years with irresistible sleep attacks. Patients with narcolepsy, especially NT1, have been found to have a high prevalence of obesity and other metabolic derangements. This narrative review aimed to address the relationship between narcolepsy and changes in weight and metabolic rate, and discuss potential mechanisms for weight gain and metabolic changes and future research agendas on this topic. This article will provide a balanced, up-to-date critical review of the current literature, and delineate areas for future research, in order to understand the pathophysiological metabolic changes in narcolepsy. Articles using predefined keywords were searched for in PubMed and Google Scholar databases, with predefined inclusion and exclusion criteria. Compared to controls, patients with narcolepsy are more likely to be obese and have higher BMIs and waist circumferences. According to recent research, weight gain in narcolepsy patients may be higher during the disease’s outset. The precise mechanisms causing this weight gain remains unknown. The available information, albeit limited, does not support differences in basal or resting metabolic rates between patients with narcolepsy and controls, other than during the time of disease onset. The evidence supporting the role of orexin in weight gain in humans with narcolepsy is still controversial, in the literature. Furthermore, the available data did not show any appreciable alterations in the levels of CSF melanin-concentrating hormone, plasma and CSF leptin, or serum growth hormone, in relation to weight gain. Other mechanisms have been proposed, including a reduction in sympathetic tone, hormonal changes, changes in eating behavior and physical activity, and genetic predisposition. The association between increased body mass index and narcolepsy is well-recognized; however, the relationship between narcolepsy and other metabolic measures, such as body fat/muscle distribution and metabolic rate independent of BMI, is not well documented, and the available evidence is inconsistent. Future longitudinal studies with larger sample sizes are needed to assess BMR in patients with narcolepsy under a standard protocol at the outset of narcolepsy, with regular follow-up.

## 1. Introduction

Narcolepsy is a chronic auto-immune sleep disorder first described in 1877 by Westphal [1]. It affects 25–50 per 100,000 individuals worldwide [2]. Usually, symptoms of narcolepsy emerge during the second decade of life; however, it has two peaks at 15 and 35 years [3]. Patients typically present with irresistible sleep attacks, sleep-related hallucinations, and sleep paralysis [3]. Excessive daytime sleepiness is the most disabling symptom and sometimes occurs in unusual circumstances like eating, talking, and driving. Moreover, in response to strong emotions, mainly positive ones like laughing, sudden loss of skeletal muscle tone may occur, which is called cataplexy [3]. Two types of narcolepsy have been described; narcolepsy type 1 (NT1), mediated by hypocretin deficiency, and mostly accompanied by cataplexy, and narcolepsy type 2 (NT2), with an absence of cataplexy [4,5].

It is believed that autoimmunity plays the primary role in causing narcolepsy. A mutation on chromosome 6 results in the human leukocyte antigen (HLA) subtype DQB1*06:02 (HLA class II allele), which leads to T-cell-mediated destruction of specific neurons in the lateral and posterior hypothalamus. These neurons produce a specific neurotransmitter known as hypocretin or orexin (a hypothalamic neuropeptide) [6]. Orexin’s name originated from the Greek word (orexis), which means appetite [7]. Orexin has two receptors, type 1 (OX1R) and type 2 (OX2R). It has been shown that orexin plays a role in controlling feeding, energy expenditure, and sleep.

**Figure 1:**
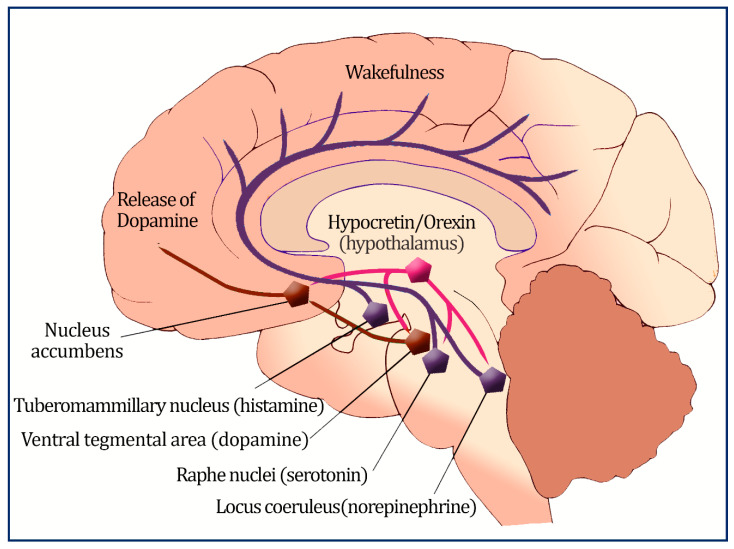
An illustration of the primary orexin neuron projections; orexin neurons located in the lateral and posterior hypothalamus regulate sleep and wakefulness by sending excitatory projections to the monoaminergic and cholinergic nuclei in the brain stem, and hypothalamic regions such as the locus ceruleus, the tuberomammillary nucleus, and the raphe nuclei.

Orexigenic neurons have projections into different brain parts, innervate many regions that enhance wakefulness, and abolish rapid eye movement (REM) sleep [8]. Orexin also connects to specific brain regions, such as the arcuate nucleus, which governs metabolism, food intake, and weight [9]; the paraventricular nucleus, the parabrachial nucleus; and the nucleus of the solitary tract, which regulates autonomic tone [10]. The primary orexin neuron projections are depicted in Figure 1. In addition, orexin and its receptors are also found scattered in the peripheral tissues like adipose tissues, the intestine, pancreas, and adrenal glands [11].

The ventral tegmental region connects orexin neurons to the reward system, as well (nucleus accumbens, containing dopamine).

In addition, metabolic diseases including obesity, hypertension, dyslipidemia, and diabetes mellitus, have been documented to complicate the course of narcolepsy, particularly NT1 [12,13,14].

This narrative review aimed to address the relationship between narcolepsy and changes in weight and metabolic rate, and discuss potential mechanisms for weight gain and metabolic changes, and future research agendas on this topic.

## 2. Search Methods

We followed published criteria about searching and reporting the literature for narrative reviews [15,16]. PubMed database and Google Scholar were searched using the subsequent keywords “Narcolepsy”, “BMI”, “eating disorder”, “eating behavior”, “metabolic rate”, “energy expenditure”, “orexin”, “hypocretin”, “leptin”, “exercise”, “ghrelin”, “diet”, and “pathophysiology”.

Inclusion criteria comprised all original articles (human and animal studies) and systematic reviews until 15 September 2022. All age groups were included. Editorials and opinions were excluded. Additionally, articles written in languages other than English were excluded.

Moreover, pertinent publications were located by looking through the references of retrieved articles (backward search) and by looking for more recent articles that cited the retrieved paper (ahead search).

For studies that addressed the aims of the review, information about the study design, development, and interventions were extracted. Each study’s data collection focused primarily on the type of intervention, goals, target population, design and conduct of the study, and the study’s findings, including participant demographics, the duration of the intervention, and the intervention’s results. In addition, we also took note of the investigators’ conclusions. The two authors independently examined each of the retrieved papers to determine eligibility and extract study data. Any discrepancies between the two authors were resolved by discussion and agreement. When enough data were available, both authors designed tables to describe the studies and summarize the results to ensure their accuracy and completeness.

## 3. Prevalence of Obesity in Patients with Narcolepsy

Numerous studies have evaluated the frequency of overweight and obesity in people with narcolepsy [17], and the literature generally confirms that body mass index (BMI) is higher in people with narcolepsy than in controls (Table 1). Waist circumference was also higher among patients with narcolepsy than among controls [18]. A systematic review and meta-analysis of 18 studies found that patients with narcolepsy had higher BMI values [18].

A study by Poli et al. reported a higher prevalence of overweight (BMI between 85th and 97th percentile) and obesity (BMI > 97th percentile) in 43 children and adolescents diagnosed with NT1, compared with the general pediatric population (78% vs. 36%, respectively) [17]. Furthermore, 60% of patients were reported to gain weight around narcolepsy onset [17]. This finding concurs with Wang et al., who reported that in 65 Chinese children with NT1 for less than a year, BMI increased considerably at six, twelve, eighteen, twenty-four, and thirty months of follow-up but not at month thirty-six [23]. In addition, Poli et al. demonstrated that high BMI was predicted by being diagnosed at a younger age, having a shorter disease duration, and lower high-density lipoprotein (HDL) levels [17]. Notably, a high prevalence of precocious puberty was reported in 17% of patients compared with obese controls [17]. Another important study supports the hypothesis of weight gain around narcolepsy onset in children recently diagnosed with NT1 [30]. A retrospective review of 30 patient records over two years before narcolepsy onset, found that overweight and obesity increased from 17% to 50% at the time of diagnosis [30]. Another recent study assessed children’s rapid weight gain (RWG) phenotype associated with narcolepsy onset [31]. The RWG narcolepsy group was younger, sleepier, and more likely to be obese at narcolepsy diagnosis than at the beginning of symptoms, which suggests a faster-moving pathological process. The RWG group still had a higher BMI z-score and a higher prevalence of obesity compared to the non-RWG group at the most recent follow-up [31].

Additionally, a reported defect in histamine neurotransmission (another wake-promoting neurotransmitter) has only been observed in the Cerebrospinal fluid (CSF) of NT1pediatric patients [32], not adult patients [33]. Since moderate obesity has been shown in knock-out mice lacking either histamine or orexin, histaminergic neurons may also be involved in the rapid weight gain process [34,35]. This may explain the higher reported incidence of weight gain in children with narcolepsy; however, more studies are needed to confirm the role of histamine in weight gain in humans with narcolepsy.

In addition, overweight/obese patients were found to have lower levels of HDL, higher systolic and diastolic blood pressure, and a higher prevalence of metabolic syndrome, which was diagnosed in 18.8% of cases, compared to a cohort of with normal BMI [30].

Case-control studies also reported a higher prevalence of overweight and obesity among adult patients with narcolepsy [36]. Schuld et al. compared patients with narcolepsy with a community-based sample and reported that the distribution of BMI in the patient sample did not substantially differ from patients who had previously received pharmacological therapy for narcolepsy and drug-naive patients [36]. Moreover, no differences were detected between healthy individuals who were HLA-DR2 positive and negative [36]. The authors concluded that an elevated BMI in patients with narcolepsy could be related to neuroendocrine abnormalities related to the condition rather than to HLA-DR2 antigen or narcolepsy medications [36], as the prepro-orexin gene is downregulated in mice with hereditary obesity due to multiple leptin system abnormalities, suggesting that orexins play a role in endocrine systems [37].

Another study in 132 adult patients with narcolepsy (mean age 47 ± 16 years) reported a prevalence of overweight (BMI 25–30) and a prevalence of obesity (BMI > 30) in 42% and 32%, respectively, compared with 24.7% and 5.2% in the general population, and 27% and 15% in 104 psychiatric inpatient controls [29]. Interestingly, BMI was not affected by the number of cataplexy attacks, symptom severity, presence of sleep paralysis, sleep-related hallucination, and medication status [29]. Nevertheless, current evidence suggests that obesity is more prevalent among NT1 patients than among NT2 patients [14,30].

In adults, longitudinal data suggest that patients with narcolepsy may continue to gain weight over time. Our team followed 32 NT1 patients for 10 years and reported an increase in BMI from baseline of 30 ± 5.1 to 33.3 ± 6 kg/m^2^ (*p* = 0.001) [38]. These results concur with a report from South Korea, after 10 years of follow-up of nine NT1 patients and nine sex and age-matched NT2 patients; BMI increased from 26.8 ± 0.9 kg/m^2^ to 29.23 ± 0.91 kg/m^2^ (*p* = 0.001) [39]. These results suggested that BMI may continue to increase in patients with narcolepsy over the years.

Compared to patients with idiopathic hypersomnia (IH), a small study demonstrated that patients with narcolepsy with low orexin had significantly higher BMI, greater waist-to-hip ratio and waist circumference, lower levels of HDL, higher total cholesterol and triglycerides, higher diastolic blood pressure, higher fasting insulin, and a higher glucose/insulin ratio (suggesting insulin resistance) [27]. Interestingly, after adjusting for BMI, patients with narcolepsy remained significantly higher in waist circumference, lower in HDL, and higher in glucose/insulin ratio compared with controls [27].

Changes in patients with narcolepsy seem to be related not only to BMI but also to fat distribution. A magnetic resonance imaging (MRI) study of 19 adolescents diagnosed with NT1, demonstrated that patients with narcolepsy had higher total abdominal adipose tissue, higher visceral adipose tissue (VAT), and higher abdominal subcutaneous adipose tissue than healthy controls of matched age and sex [22]. On the other hand, another study reported no difference between 14 patients with narcolepsy and 14 age-sex-BMI-matched controls in terms of body fat percentage, fat mass, fat mass index, fat-free mass, fat-free mass index, and total body water [40]. However, less muscle mass was found in patients with narcolepsy compared with controls [40].

Putting it all together, overweight and obesity are prevalent and well-documented in patients with narcolepsy across different ages. Based on the currently available evidence, it seems that the early onset of narcolepsy is associated with an increase in BMI [17,30]. The coincidence of narcolepsy onset and orexin deficiency in children and adolescents may produce amplified weight gain, possibly due to the reduced activities associated with sleepiness and reduced metabolic rate [23]. Nevertheless, weight gain has been reported in adult patients with narcolepsy too. Therefore, closely monitoring overweight and obesity, and applying appropriate weight reduction strategies are crucially important. Moreover, generating a specific guideline for patient follow-ups, including the assessment of related laboratory investigations (such as lipid and glucose panels) at certain time intervals, may significantly impact decreasing obesity-related cardiometabolic risks and improve patients’ quality of life. Additionally, studies are needed to characterize patients with narcolepsy who are more vulnerable to developing obesity and the time period of the illness that carries the higher risk of gaining weight.

## 4. Proposed Theories of Weight Gain in Narcolepsy

**Figure 2:**
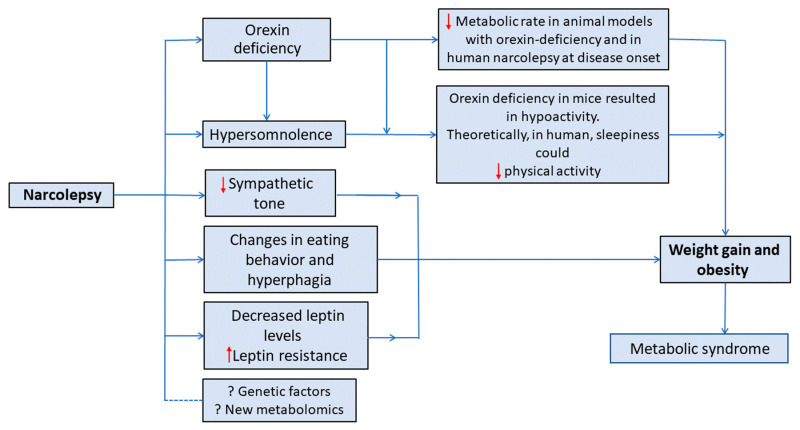
A summary of the proposed mechanisms for increased weight in patients with narcolepsy.

The mechanism behind weight gain in narcolepsy is not fully understood. In fact, obesity in patients with narcolepsy may be caused by a variety of causes, such as: less physical activity [41], binge eating behavior [42], low metabolic rate [23,42], reduced sympathetic tones [43], growth hormone deficiency [44], and reduced plasma leptin level [45]. Nevertheless, no consistent answer has been concluded, as investigating these possible contributing factors has shown conflicting results. Figure 2 presents a summary of the proposed mechanisms for increased weight in patients with narcolepsy.

### 4.1. Orexin’s Role in Metabolism

Orexin modulates calorie intake, energy consumption, and sleep; in response to metabolic signals such as peripheral blood glucose, leptin, and ghrelin levels, the neurons that produce orexin and quickly assess the body’s nutritional condition [46]. Several studies in rats showed that intracerebroventricular injection of orexin (mainly orexin-A) in pharmacological doses increases food intake [47,48,49,50]. In contrast, in rats, intracerebroventricular injection of orexin receptor antagonists or antibodies decreases food consumption [47,48,49,50]. Orexin enhances food-seeking behavior in rats, and eating results in decreased orexin levels and low activity of hypocretinergic neurons [51]. Moreover, diurnal fasting increases orexin levels in humans [52].

Furthermore, it has been reported that orexin-A injection improved the mice’s basal metabolic rate (BMR) without the need for physical exercise [53]. Additionally, orexin is a crucial central neuropeptide controlling non-exercise activity thermogenesis [54]. Available results indicate that in male Sprague-Dawley rats, dual orexin receptor antagonists lower orexin-A-induced increases in spontaneous physical activity, total energy expenditure, and non-exercise activity thermogenesis during spontaneous physical activity, waking, rest, and sleep [55]. Recent human studies also suggest that the orexin receptor antagonist “Suvorexant” may affect metabolism. In a study by Nakamura and Nagamine on children with insomnia who were started on the anti-orexin suvorexant, fasting insulin levels at week 8 were lower than baseline, nevertheless failed to achieve statistical significance, indicating that suvorexant at the therapeutic dose for insomnia may have beneficial effects on metabolism [56]. Another study assessed the chronotherapeutic efficacy of suvorexant on subjective sleep parameters and metabolic parameters in patients with type 2 diabetes and insomnia, and demonstrated that abdominal circumference and daily sucrose intake were significantly decreased [57]. A third short-term study demonstrated that 7 days of suvorexant improved daily glycemic control in patients with type II diabetes, which was coupled with changes in sympathomimetic tone and enhanced insulin sensitivity; however, anthropometric data were not reported [58].

Orexin also controls brown adipose tissue thermogenesis and increases energy expenditure by improving non-exercise activity thermogenesis [59]. It has been demonstrated that orexin injections increase brown adipose tissue, CO_2_ generation, and thermogenesis in rats [59]. In mice, orexins also delay the onset of diet-induced obesity by raising the sensitivity of orexin-coupled hypothalamic neurons and concurrently elevating nonesterified fatty acids and white adipose tissue levels of lipolysis [60].

Due to these facts, the loss of orexin, in patients with narcolepsy, is logically expected to result in weight loss and hypophagia. Yet, surprisingly, obesity and overweight are highly prevalent and well-documented in patients with narcolepsy [29,61].

### 4.2. Orexin and Eating Behavior

It is hypothesized that orexigenic neuron degeneration affects the metabolic profile in different ways, resulting in binge eating behavior in a group of patients [62] and hypophagia in others [42]; nevertheless, the evidence regarding this hypothesis is controversial in the literature.

Through orexigenic neurons of the hypothalamus projections, the ventrotegmental area of the midbrain mediates reward-seeking behaviors [63]. Also, the dorsomedial and paraventricular hypothalamic regions mediate food-seeking behavior through thyrotropin-releasing hormone (TRH) and corticotropin [63]. Moreover, it is suggested that through the melanocortin pathway, orexin deficiency causes binge eating [64]. Current evidence suggests that the prevalence of eating disorders, including bulimia and binge-eating disorders, is higher in patients with narcolepsy [65,66,67]. Interestingly, rising BMI levels trigger eating disorders like bulimia [42], which are linked to worsening symptoms of daytime sleepiness [68].

A study used functional MRI in NT1 orexin-deficient patients to clarify the function of orexin in the neurocognitive processes generating food attentional bias and reported an increase in ventral-medial prefrontal cortex activity during food-driven attention, compared to controls [69]. The finding that neurocognitive pathways influence NT1 patients’ processing of food cues suggests aberrant motivational brain responses to food in a condition of orexin deficit that may lead to overeating in this illness.

Studies showing a higher incidence of binge eating in patients with narcolepsy appear to be at odds with the theory that abnormal eating and sleep in binge eating disorders are associated with higher orexin activity [70]. Using binge eating as a behavioral intervention to lessen tiredness and prevent disruptive sleep episodes may explain this phenomenon [66]. A different, developmental stage-based notion by Barson contends that postnatal orexin cell loss decreases food intake while adult orexin cell loss enhances it [71]. Additionally, the overeating and obesity phenotypes in adult orexin cell-knockout mice suggest that adult orexin cell loss may result in binge eating-like behavior and weight increase [72]. Further, another study that involved the loss of orexin cells in mice between weeks 1 and 8 of age found that the loss of these cells resulted in a nearly 30% reduction in food intake [73]. This is supported by the fact that the typical age of onset of narcolepsy is around the late second and early third decades [74].

However, in humans, a report examining 116 patients with narcolepsy and 80 controls failed to find elevated rates of binge eating-like behavior in patients with narcolepsy [75]. Additionally, a recent study used the Eating Disorder Evaluation Questionnaire 6th edition (EDE-Q), which is a self-report version of the Eating Disorder Examination that evaluates characteristics of eating disorders and makes four subscale results: dietary restraint, eating concern, weight concern, and shape concern [76]. The EDE-Q total score did not differ between NT1, NT2, and controls [76]. The above reports indicate the complexity of the association, and suggest the existence of different individual phenotypes of narcolepsy, calling for more research to explore the link between loss of orexin in patients with narcolepsy and metabolic and eating disorders.

### 4.3. Leptin, Ghrelin, and Other Hormonal Changes

The proteohormone leptin, an *obese* gene product, is secreted by adipocytes to regulate body fat mass [28]. The orexinergic system communicates with the hypothalamus network that responds to leptin. Orexin and leptin can stimulate and inhibit leptin-responsive cells [28]. Leptin and orexin levels vary inversely in fasting subjects [52,77]. Ghrelin is an appetite-stimulating peptide that functions as a peripheral orexigen that blocks leptin’s effects [78]. It has been suggested that the observed overweight in patients with narcolepsy may be partially attributed to changes in leptin and ghrelin levels.

A study that measured peripheral leptin levels in 42 patients with narcolepsy and 31 BMI-matched controls reported no reduction in peripheral leptin levels in patients with narcolepsy [79]. A subsequent study reported no changes in the mean 24 h total plasma ghrelin and leptin levels or food-induced suppression of ghrelin concentrations between patients with narcolepsy and healthy controls [80]. A third study showed no difference in serum and CSF leptin levels between NT1 patients and controls [28].

A study that sought to find the association between BMI, orexin, and leptin levels in NT1 patients divided into low orexin levels (26 cases) and normal orexin levels (23 cases), and compared them with 46 healthy controls matched for sex and age [81], reported a statistically significant increase in the number of obese patients (BMI > 30) in narcolepsy compared with controls. Nonetheless, the mean BMI did not differ significantly between groups. In addition, comparing the two groups of patients with narcolepsy with normal and low orexin levels, showed no statistically significant difference in the mean BMI, which suggests that mechanisms other than orexin deficiency are also involved in bodyweight changes. Furthermore, no difference in the leptin/BMI ratio was reported in patients with normal and low orexin and controls [81], indicating a lower role for orexin on leptin. Another study found no difference in the fasting and post-prandial ghrelin levels between eight patients with narcolepsy and matched controls [82]. On the other hand, a study reported a 50% reduction in 24 h mean plasma leptin levels in six orexin-deficient narcoleptics, compared to controls matched for fat mass, BMI, waist-to-hip ratio, age, and sex [83]. Interestingly, the normal nocturnal acrophase of plasma leptin levels observed in controls was absent in patients with narcolepsy, suggesting a possible circadian rhythm disturbance of leptin secretion in narcolepsy [83]. However, the study’s power was low because of the small sample size. A subsequent study on 38 patients diagnosed with NT1 reported that NT1 patients had significantly elevated CSF leptin levels compared to ethnically matched controls [84]. Furthermore, the CSF leptin levels positively correlated with normalized BMI, and its high values may indicate leptin resistance [84]. Nonetheless, serum leptin levels were not measured, and BMI was not matched among the two groups.

In conclusion, most of the current evidence does not support a role for leptin and ghrelin secretion in weight gain in patients with narcolepsy. The timing of sample collection and its relation to mealtimes may cause dissimilarities between different studies. Moreover, it is important to note that narcolepsy is a chronic disease, and compensatory mechanisms may develop over time [38,80], so investigating patients with narcolepsy in the chronic phase only, without assessing changes associated with disease onset, may generate inaccurate conclusions regarding orexin’s effect on leptin and ghrelin, and hence body weight. In addition, measuring leptin and ghrelin levels following a standard research protocol, with fixed bedtime and determined meal components and times, may not be illustrative of the patient’s real life, as leptin and ghrelin secretion is affected by several factors.

Other hormonal mechanisms have been proposed too. Animal studies have suggested that orexin deficiency increases insulin resistance. Orexin reduces insulin resistance and endoplasmic reticulum stress in the mouse liver [85]. Furthermore, through the downregulation of insulin receptors and disruption of intracellular insulin receptor signaling, orexin deficit causes insulin resistance [86,87]. These results could explain why patients with narcolepsy had plasma insulin levels that were greater than those of controls. However, in humans, glucose tolerance tests of patients with narcolepsy did not substantially vary from those of the general population when controlled for BMI as a potential confounding factor [88].

Moreover, growth hormone (GH) response to clonidine and arginine tests, showed lower levels of GH response, below the deficiency level (8 ng/mL), suggesting that GH secretion may be altered due to BMI changes in narcolepsy [30].

### 4.4. Reduced Sympathetic Tone

In a study of in vitro rat brain slice preparations, when orexin was administered or orexin-secreting neurons were stimulated, sympathetic output measures, including heart rate, blood pressure, and body temperature increased [89]. Conversely, orexin neuron-ablated and orexin-defective mice showed reduced heart rate and blood pressure during wakefulness [90,91]. This concurs with the Sorensen et al. findings of reduced heart rate following arousals in NT1 patients, supporting the hypothesis that orexin deficiency decreases sympathetic tone, which might explain why narcolepsy is associated with obesity and a possible lower metabolic rate [92]. In real life, it is not that easy. Studies on sleep in transgenic and knock-out mice yielded contradictory outcomes, where some studies reported a rise in blood pressure during sleep [93]. Moreover, other human studies suggested that sympathetic activation in narcolepsy, reflected by a rise in heart rate during wakefulness and sleep, and the physiological blood pressure dipping associated with sleep appeared to be attenuated in patients with narcolepsy [94]. Therefore, future cardiovascular research utilizing state-dependent analyses may be helpful to better understand the direct impacts of orexin deficiency on sympathetic tone.

### 4.5. The Role of Diet and Dietary Components

Several reports have assessed the composition and size of meals eaten by patients with narcolepsy, as changes in meals and their content may contribute to overweight and obesity in patients with narcolepsy [95]. An earlier review of the dietary histories of spontaneous food choices in 12 patients diagnosed with NT1 reported reduced carbohydrate and food intake compared with controls, supporting the theory of hypophagia in narcoleptics due to low orexin levels [95]. However, recall bias cannot be ruled out, and BMIs were not matched between cases and controls. These results were replicated in a subsequent study, as 14 patients with narcolepsy had significantly lower food intake when assessing their 3-day caloric intake compared to 14 patients diagnosed with IH; there was no difference in the type of food consumed [27]. Pollak and Green reported no differences in the frequency, number, size, or content of meals in six free-running narcoleptics compared to controls, excluding eating disorders in narcolepsy subjects [62].

Although following a healthy diet can lead to weight loss, seven patients with narcolepsy reported improvements in daytime sleepiness, sleep attacks, and sleep paralysis after eight weeks of adherence to a low carbohydrates ketogenic diet [96]. Relatively lower blood glucose levels compared to baseline may have contributed to the activation of orexigenic neurons and improvements in daytime sleepiness in these patients [96,97]. This study highlights the importance of a healthy diet for patients with narcolepsy not only to lose weight but also improve other narcolepsy symptoms [96].

### 4.6. Physical Activity

Reduced physical activity could be a cause of high BMI in narcolepsy, as ablation of orexin neurons in mice resulted in hypoactivity and significant weight gain without an increase in food or drink intake [98]. Weight gain and hypoactivity co-occur in narcolepsy animal models [98,99], which raises the possibility that weight gain is the result of decreased energy expenditure, possibly secondary to a reduction in motivated behavior. Additionally, it is possible theoretically that hypersomnolence in patients with narcolepsy results in a reduction in physical activity.

To support this finding in humans, Bornstein et al. used an armband accelerometer sensor to assess one-week physical activity in 53 NT1 patients compared to 23 patients diagnosed with either NT2 or IH [100]. Unexpectedly, there was no difference in physical activity between the two groups, despite the reported higher BMI in NT1 patients. Moreover, a subgroup analysis detected no difference in physical activity between patients with decreased and normal orexin levels [100]. However, no control group of healthy volunteers was compared with patients, and a retrospective assessment of physical activity for a limited duration of one week could not provide a definitive conclusion in this regard. Another study that explored the importance of implementing physical activities in children with narcolepsy, reported that patients engaged in leisure time physical activity had lower BMI, longer night sleeping hours, less daytime sleepiness, better sleep efficiency, and decreased number of naps than patients with narcolepsy of a sedentary lifestyle [101]. Larger case-control studies in all age groups of patients with narcolepsy are needed to assess the association between physical activity and weight gain.

### 4.7. Genetics Factors

First-degree relatives of patients with narcolepsy have been found to have higher BMI compared to the general population, suggesting that genetic traits contributed to this finding, even if an association with HLA-DR2 antigen was not found [29,36].

Schuld et al. sought to answer the question of whether obesity in NT1 patients is caused by the disease itself, or by genetic pre-morbid factors. Therefore, they investigated the possible association between the HLA-DR2 antigen, which is strongly associated with narcolepsy, and BMI in 117 HLA-typed healthy males. However, the results indicated that obesity was not associated with HLA-DR2 antigen, as higher BMI was not found in DR2 antigen-positive individuals compared to the negative group [36]. On the same line, a recent study demonstrated that the HLA-DR2 antigen was not associated with metabolic-syndrome-related disorders in NT1 patient [14].

Further case-control studies among HLA-typed matched groups are needed to prove this finding.

### 4.8. Metabolomics

A recent study compared the plasma metabolic profiles (assessed 141 circulating, low-molecular-weight metabolites) of patients with NT1 with controls, according to their BMI status in drug-free fasting plasma samples from 117 patients with NT1 (including 41 obese people) and 116 BMI-matched controls [102]. The findings indicated that glutamate, sarcosine, serotonin, tryptophan, nonaylcarnitine, and a few phosphatidylcholines are some metabolic diagnostic biomarkers for NT1. These findings are interesting and, if confirmed in future studies, might provide new ideas for understanding the pathophysiology of obesity in patients with narcolepsy and help develop new treatment modalities for obesity in narcolepsy.

Based on the above, we hypothesize that the high prevalence of overweight and obesity in patients with narcolepsy is multifactorial, and different patients phenotypes may contribute to this finding. Hypersomnolence associated with narcolepsy could reduce physical activity in a group of patients, leading to a sedentary lifestyle and weight gain. Another group of patients with narcolepsy may binge eat and ingest more snacks to overcome the effect of sleepiness. Furthermore, a subgroup of patients with narcolepsy might have a reduced metabolic rate accompanying disease onset or later during the disease course, which could precipitate weight gain in this group. Although no genetic factors have been identified, further research may suggest a role for specific genes causing obesity in narcolepsy. However, the role of leptin and ghrelin cannot be neglected, and newly discovered metabolomics may provide future understanding regarding the mechanisms behind weight gain in narcolepsy.

## 5. Changes in Metabolic Rate (Energy Expenditure) in Patients with Narcolepsy

It has been reported that preadipocytes in the brown adipose tissue of animal models with orexin deficiency may become incapable of differentiating, which in turn reduces thermogenesis and energy expenditure [103]. Orexin has also been demonstrated to control the metabolism of muscle glucose via the activation of muscle sympathetic neurons and beta(2)-adrenergic transmission [87]. Moreover, orexin receptor-2-deficient mice revealed lower energy expenditure when fed a high-fat diet [104]. Therefore, it has been proposed that orexin may cause a lower metabolic rate in some patients with narcolepsy resulting in obesity, despite eating fewer calories.

Basal energy expenditure (BEE) (or basal metabolic rate (BMR)) is the required energy to maintain the basic metabolic functions of the cells and organs in the absence of physical activities, psychological stress, and recent food consumption. In clinical settings, indirect calorimetry (IC) is considered the gold standard for measuring the BEE, and individuals preparing for IC should follow strict instructions, including fasting for at least 5 h, abstaining from physical activities, nicotine, smoking, alcohol, and caffeine for specific hours before measurement [105,106].

Several studies have investigated metabolic rate in patients with narcolepsy, BMR or resting metabolic rate (RMR), which could contribute to the increased prevalence of obesity in these patients. Indeed, the studies showed inconsistent results; few studies revealed reduced BMR-RMR, while others showed normal ones (Table 2) [23,40,42,43,107]. The dissimilarities between these results could be related to differences in the assessment methods used, including the absence of a standardized protocol for performing indirect calorimetry, unmatched BMI scope, studying patients at variable periods of the disease course, and not documenting orexin levels in all studies. Additionally, most studies comprised a small sample size.

A longitudinal study in China assessed the BMR and BMI in children recently diagnosed with NT1 [23]. The investigators followed the patients every six months for 36 months, and compared them with age-matched control peers while controlling for caloric intake and physical activity. The patient group had a significant increase in BMI on follow-up visits at six, 12, 18, 24, and 30 months, but not at 36 months. In addition, the BMR, measured by indirect calorimetry, showed a 25% reduction compared with healthy controls at a follow-up of 6 months, but not at 36 months. Although this study was done on a small sample (*n* = 18), and orexin levels were not measured in all patients, it revealed lower BMR in patients with narcolepsy at disease onset (first 6 months) of narcolepsy, which concurs with the propensity to gain weight at diseases onset. BMR was normal and similar among the two groups after three years, which could suggest a role for the lower BMR in causing weight gain around disease onset in narcolepsy, which is supported by a cessation in BMI increase at 36 months [23]. These findings endorse what was found in 1934 when Daniels reported substantial weight gain (5–45 kg) around disease onset in half of the patients with narcolepsy [108].

In another study in France; Chabas et al. measured RMR using indirect calorimetry and urinary nitrogen production in adult patients with narcolepsy and reported a lower RMR when compared with controls matched for age, sex, and ethnicity [42]. Furthermore, patients with NT1 tended to eat less than controls, which was interpreted as secondary to a low orexin level accompanying NT1 [42]. It is important to highlight that those who were overweight in the control group also had low RMR [42]. However, as the study was conducted with a small sample size (*n* = 13), the study’s power was low, raising the error margin risk. Furthermore, the orexin level was not assessed in patients. In addition, the BMI was not matched among the two groups, which could make the results of lower RMR in patients with narcolepsy not comparable with controls who had normal BMI [42].

On the other hand, a study compared BMR measured by indirect calorimetry in two groups with matched age and BMI (13 patients with narcolepsy and 30 controls) and revealed no significant differences in BMR between the two groups [107]. However, a subgroup analysis illustrated lower BMR in non-obese patients with narcolepsy compared to the BMI-matched controls. This finding suggests that narcolepsy lowers the set point of BMR around disease onset, causing an increase in BMI set point, and after a higher BMI is reached, BMR abnormalities do not occur [107]. These findings support the results of Fronczek et al., which showed no significant differences in RMR in 15 patients with narcolepsy, compared to 15 controls of matched BMI [43]. In addition, no differences in the respiratory quotient (RQ) (VCO2/VO2), oxygen consumption, carbon dioxide production, and fat or carbohydrate substrate combustion were detected among the two groups [43].

In line with previous studies and using indirect calorimetry, our team found no difference between 14 patients with narcolepsy and 14 age-sex-BMI matched controls in terms of RMR [40]. Furthermore, the RMR of the NT1 and NT2 subgroups did not significantly differ [40]. However, we found that patients with narcolepsy had a significantly lower RQ. An elevated RQ could suggest future fat gain because RQ is considered a measure of substrate oxidation (greater values indicate higher carbohydrate vs. fat oxidation) [109]. These findings imply that during periods of fasting, the metabolic systems of patients with narcolepsy used more fat and fewer carbs than the controls (RQ was in the range of fat utilization, and the percentage of utilized fat was much greater). The greater proportion of fat oxidation and lower carbohydrate consumption may imply a state of insulin resistance, which is typical of the metabolic system of patients with narcolepsy, even at an early age [109].

A recent systematic review investigated metabolic profiles in narcolepsy; four studies that measured BMR-RMR were assessed and found no statistically significant difference in the BMR-RMR between patients with narcolepsy (*n* = 53) and controls (*n* = 75) [18]. Nonetheless, a meta-analysis was not performed because of the small number of studies.

In summary, measuring metabolic rate may lead to substantial insights into the pathophysiology of obesity in patients with narcolepsy. However, the limited number of studies evaluating metabolic rate in the literature and the disagreement about its reduction in patients with confirmed low orexin levels makes an association between the development of obesity and metabolic rate reduction in narcolepsy less likely. To confirm this, it would be helpful to establish a standard protocol for measuring BMR from the onset of narcolepsy, with regular follow-ups of BMR and BMI in a large number of patients compared with controls matched for BMI, age, and sex, and in this regard, further research may be of additional value.

## 6. Narcolepsy Treatment and Weight Loss

Sodium oxybate (SXB), the sodium salt of gamma-hydroxybutyrate, is approved for the treatment of narcolepsy with cataplexy at seven years of age and older [110]. Several reports have evaluated the effect of SXB on weight loss in patients with narcolepsy; at one-year follow-up of 30 NT1 children treated with SXB, overweight and obesity dropped from 25.71% to 17.14%, and 31.42% to 17.14%, respectively, suggesting that SXB induce weight loss in narcolepsy [30]. These findings were replicated in a subsequent study, as 129 NT1 children treated with SXB experienced significant weight loss at a one-year follow-up [110]. Another study on 54 adult patients with narcolepsy (mean age 48.3 years) treated with SXB found a significant weight loss of 5 kg (kgs) in individuals with cataplexy, with no significant weight loss in patients with narcolepsy without cataplexy [111]. In addition, a maximum weight loss of 30 kgs was reported, with no significant correlation between the dose of SXB or duration of treatment and weight change [111]. SXB may promote weight loss in narcolepsy by improvement of daytime sleepiness and enhancing alertness, consolidating nighttime sleep, and increasing deep sleep with possibly subsequent normalization of growth hormone secretion [44,111]. Another stimulant medication named lisdexamfetamine (a central nervous system stimulant) is used off-label to treat narcolepsy [112]. Weight loss was also observed in four of five patients with narcolepsy treated with lisdexamfetamine [112].

Furthermore, Pitolisant is an H3 histamine receptor antagonist/inverse agonist indicated for the treatment of narcolepsy with or without cataplexy [113]. One study reported that obese mice lost a slight amount of weight after 14 days of pitolisant administration [114]. However, in a systematic review, weight gain was reported as a possible adverse event of pitolisant, observed in 2.9% of 342 patients with narcolepsy [113].

In animal models, it has been suggested that OX2R signaling encourages energy expenditure through leptin sensitization; moreover, it changes the hypothalamic set-point that regulates metabolism, food intake, leptin sensitivity, and insulin sensitivity [115]. Theoretically, therapeutic agonists targeting OX2R in patients with narcolepsy may be able to prevent or reverse weight and metabolic changes in this group of patients. The first OX2R-selective agonist, danavorexton, is still being studied in patients with NT1 and other hypersomnia disorders (ClinicalTrials.gov Identifiers: NCT03332784 and NCT03748979 in patients with narcolepsy). However, in animal models, a recent study demonstrated that 14-day of continuous treatment with danavorexton diminished weight gain in orexin/ataxin-3 mice, a mouse model of NT1 [116]. Additionally, danavorexton marginally, but significantly, decreased total calorie intake. To clarify the mechanisms underlying the anti-obesity action of OX2R agonists in NT1 animal models, additional research is required. Additionally, human data on the effect of OX2R-selective agonists on weight gain, feeding, and metabolic rate in patients with narcolepsy are lacking.

In conclusion, the discovery of altered BMI and weight in patients with narcolepsy receiving medication such as sodium oxybate [30,110,111] and lisdexamfetamine [112], opens up new research opportunities for the investigation of possible novel mechanisms behind weight increase in patients with narcolepsy.

## 7. Conclusions and Future Directions

Obesity and increased BMI and waist circumference are more prevalent among patients with narcolepsy than controls. Current evidence suggests that weight gain in narcolepsy may be higher during early disease onset. However, the exact mechanisms of this weight gain are not known. Current evidence, though limited, does not support changes in BMR and RMR in patients with narcolepsy compared with controls, except at disease onset. Moreover, current evidence did not document significant changes in different hormonal profiles related to weight gain, such as serum GH, plasma and CSF leptin, and CSF melanin-concentrating hormone levels.

Nevertheless, more work is needed to characterize the metabolic effects of orexin, including regulation of food intake with its relation to ghrelin and leptin hormones, fat and glucose metabolism, autonomic control, and energy homeostasis. Additionally, future longitudinal studies with larger sample sizes are needed to assess BMR in patients with narcolepsy, under a standard protocol at the outset of narcolepsy, with regular follow-ups of BMR and BMI compared to controls matched for BMI, age, and sex. Furthermore, clustering patients into different phenotypes may significantly impact our understanding of narcolepsy-related obesity.

Meanwhile, early screening for overweight and obesity in patients with narcolepsy is crucially valuable, as early non-pharmacological and pharmacological interventions could overcome the weight gain accompanying narcolepsy and reduce obesity-related complications. Patients with narcolepsy should receive proper health education about obesity and its effects. In addition, advice for a healthy lifestyle, including a healthy diet and physical exercises, could be implemented early in the management plan. Furthermore, following patients with narcolepsy in the clinic may include a periodic assessment of increased BMI and an investigation of metabolic derangements.

## Figures and Tables

**Table 1:** A summary of the studies that assessed the prevalence of overweight and obesity in patients with narcolepsy.

Study/Year Country	Study Design	No of Cases	Prevalence of Overweight (OW), Obesity (OB)%, or Mean BMI ± SD in Cases	No of Controls	Prevalence of Overweight (OW), Obesity (OB)%, or Mean BMI ± SD in Controls	*p*-Value
Filardi [19], 2020, Italy	Case-control	38	OW 28.95 OB 39.47	21	OW 4.76 OB 28.57	<0.05
Barateau [20], 2019, France	Case-control	92	OW 38.04 OB 23.91	109	OW 27.52 OB 4.59	<0.000
Vandi [21], 2019, Italy	Case-control	27	OW 62.96 OB 18.5	19	OW 21.1 OB 21.1	0.010
Drissi [22], 2018, Sweden	Case-control	19	24.72 ± 6.37	17	21.22 ± 2.42	0.039
Wang [23], 2016, China	Case-control	65	OW 26.15 OB 38.46	79	OW 5.06 OB 3.80	0.002 <0.001
Kovalska [24], 2016, Czech Republic	Case-control	42	31.47 ± 5.41	46	27.53 ± 6.11	0.0021
Donadio [25], 2014, Italy	Case-control	19	25 ± 4	19	23 ± 3	<0.05
Dauvillers [26], 2012, France	Case-control	50	BMI 25.11 [17.01–38.30]	42	BMI 21.40 [18.30–29.10]	0.0001
Poli [27],2009, Italy	Case-control	14	28 ± 4.4	14	24.2 ± 2.8	0.012
Arnulf [28], 2006, France	Case-control	93	27.6 ± 0.6	111	25.0 ± 0.4	<0.05
Dahmen [29], 2001, Germany	Case-control	132	28.2 ± 5.5	104	24.5 ± 4.7	<0.0001

**Table 2:** A summary of the studies that compared metabolic rates in patients with narcolepsy and controls.

Study/Year Country	Study Design	No of Cases	Mean Age of Cases	No of Controls	Mean Age of Controls	BMR/RMR
Abulmeaty [40], 2020, Saudi Arabia	Case-control	7	25.29	14	27.86	RMR; No significant difference
Wang [23], 2016, China	Case-control	18	13.75	16	14.03	BMR; Less by 25% in cases at 6 months follow up/normal at 36 months
Dahmen [107], 2009, Germany	Case-control	13	36.54	30	36.37	BMR; No significant difference. Lower BMR was detected in non-obese cases compared with controls
Fronczek [43], 2008, The Netherlands	Case-control	15	36.3	15	35.6	RMR; No significant difference
Chabas [42], 2007, France	Case-control	13	26.25	9	29	RMR was less in cases compared with controls
